# Estimating malaria parasite density: assumed white blood cell count of 10,000/μl of blood is appropriate measure in Central Ghana

**DOI:** 10.1186/1475-2875-11-238

**Published:** 2012-07-23

**Authors:** Dennis Adu-Gyasi, Mohammed Adams, Sabastina Amoako, Emmanuel Mahama, Maxwell Nsoh, Seeba Amenga-Etego, Frank Baiden, Kwaku Poku Asante, Sam Newton, Seth Owusu-Agyei

**Affiliations:** 1 Kintampo Health Research Centre (KHRC), Box 200, Kintampo North, B/A, Ghana

## Abstract

**Background:**

White blood cells count (WBCc) is a bedrock in the estimation of malaria parasite density in malaria field trials, interventions and patient management. White blood cells are indirectly and relatively used in microscopy to estimate the density of malaria parasite infections. Due to frequent lack of facilities in some malaria-endemic countries, in order to quantify WBCc of patients, an assumed WBCc of 8.0 X 10(9)/L has been set by the World Health Organization to help in estimating malaria parasite densities.

**Methods:**

This comparative analysis study, in Central Ghana, compiled laboratory data of 5,902 *Plasmodium falciparum* malaria parasite positive samples. Samples were obtained from consented participants of age groups less than five years. Full blood counts (FBC) of participants’ samples were analysed using the ABX Micros 60 Haematology Analyzer. Blood slides were read by two competent microscopists to produce concordant results. All internal and external quality control measures were carried out appropriately. Parasite densities were calculated using participants’ absolute WBCc and assumed WBCc of 5,000 to 10,000 per microlitre of blood.

**Results:**

From the 5,902 Pf malaria positive samples, the mean (SD) WBCc and geometric mean parasite density were 10.4 (4.6) × 10(9)/L and 7,557/μL (95 % CI 7,144/μL to 7,994/μL) respectively. The difference in the geometric mean parasite densities calculated using absolute WBCs and compared to densities with assumed WBCs counts were significantly lower for 5.0 × 10(9)/L; 3,937/μL, 6.0 × 10(9)/L; 4,725/μL and 8.0 × 10(9)/L; 6,300/μL. However, the difference in geometric mean parasite density, 7,874/μL (95 % CI, 7,445/μL to 8,328/μL), with assumed WBCc of 10.0 × 10(9)/L was not significant.

**Conclusion:**

Using the assumed WBCc of 8.0 X 10(9)/L or lower to estimate malaria parasite densities in Pf infected children less than five years old could result in significant underestimation of parasite burden. Assumed WBCc of 10.0 × 10(9)/L at 95 % CI of geometric mean of parasite density statistically agreed with the parasite densities produce by the absolute WBCc of participants. The study suggests where resources are limited, use of assumed WBCc of 10.0 × 10(9)/L of blood to estimate malaria parasite density in central Ghana. Preferably, absolute WBCc should be used in drug efficacy and vaccine trials.

## Background

Each year approximately300–500 million malaria infections lead to over one million deaths, of which over 75 % occur in African children aged under five years infected with *Plasmodium falciparum*[1]. About 40 % of the world’s children live in malaria-endemic countries [2,3].

White blood cells (WBC) play a vital role in the body's immune defence against disease. The number of WBC may be reduced or increased depending on a disease condition or a reaction occurring in the body [4]. Such reactions could be normal or abnormal [4]. WBCs are indirectly and relatively used in estimating *Plasmodium* parasitaemia by counting, on microscopic examination of Giemsa-stained blood smears, the number of parasites against a predetermined number of WBC. Microscopy is routinely relied upon as a primary endpoint measurement for epidemiological studies, intervention studies, and clinical trials [5].

Complete blood counts, particularly WBC counts, can be performed with new generation automated haematology analyzers [6], and/or manually using stains, the microscope and the Neubauer chamber and counters [7,8] though the accuracy of the later method is more subjective. With the cost involved in the purchase and use of fully automated or semi-automated haematology analyzers, some facilities frequently have to fall back on the manual methods to determine the complete blood count of a patient for management. Another burden is the cost of automated systems to ensure their prompt validation by constant servicing, maintenance and implementation of rigorous quality systems.

WBC are critically important in malaria intervention studies and patient management. Due to the frequent lack of facilities in some malaria-endemic countries to quantify WBCc of patients, an assumed WBCc of 8.0 X 10(9)/L [9], set by the World Health Organization (WHO), is conventionally used to estimate malaria parasite densities. Quantification of malaria parasites yields clinically useful information in patient management and drug efficacy studies [10]. Using the conventional method to quantify parasite densities may generate systematic errors and lead to incorrect conclusions [4,11].

The use of assumed WBCc rather than the absolute WBCc could lead to over-estimation of the parasite density in an infection [2] or *vice versa* depending on several factors, such as burden of malaria and other infections, the acceptable reference value for WBC in the area and the variability in the techniques adopted by microscope blood slide readers [5].

The study therefore compared the parasite densities calculated with the absolute and assumed WBCc of participants enrolled in malaria studies in Central Ghana to assess the impact of using assumed WBCc to estimate parasitaemia of *P. falciparum* infections.

## Methods

### Site description

The study was carried out on laboratory data from the Kintampo North Municipal and South District of Ghana, which cover in total, an area of 6,883 sq km with a population of approximately 189,958, and also from Wenchi Municipality and Tain District, which cover an area of 7,619 km^2^ with an approximate population of 166,641 (Ghana Districts). The study area is located within the forest-savannah, transitional ecological zone in Ghana. Studies in the area showed 50 % prevalence of malaria parasitaemia among children less than 10 years of age (symptomatic/asymptomatic). Malaria transmission is perennial, with entomological inoculation rate of 269 infective bites per person per year [12]. *Plasmodium falciparum* malaria predominates in this area with seldom malaria resulting from *Plasmodium malariae* and *Plasmodium ovale*. Mean monthly temperatures range between 18 °C and 38 °C and rainfall averages 1,250 mm per annum for the sites selected, making conditions optimal for vector abundance*. Plasmodium falciparum* resistance to chloroquine was greater than 40.0 %, similar to that recorded in other parts of the country [13]. Malaria microscopy is available for malaria diagnosis in hospitals in the selected site. Malaria rapid diagnostic test (RDT) kits are supposed to be used in peripheral health centres. Anti-malarials, particularly ACT, for uncomplicated malaria are registered to be sold over the counter in the study area and other parts of Ghana.

### Description of study participants

The data analysis was carried out on laboratory results obtained from all children under five years old, recruited into three major malaria studies carried out in KHRC for the period of April 2009 to March 2011. Children less than five years of age were selected because they are the most vulnerable group and represent the population mostly recruited in the majority of clinical trials of anti-malarials and malarial vaccines in sub-Saharan Africa. The children ranged from day-old babies to those aged five years depending on the design of the various studies.

### Ethics

The studies adopted were approved by the ethical review committees of the Ghana Health Service, London School of Hygiene and Tropical Medicine, KHRC, Noghuchi Memorial Institute of Medical Research and The Food and Drugs Board, Ghana. Community entry involved explaining the studies to key community opinion leaders followed by community durbars/meetings. At these meetings, the study aims, objectives, risks and benefits were explained to all participants and informed consent was sought from all mothers whose children participated in the studies. All data collected were kept in locked cabinets to ensure confidentiality.

### Blood sample collection

Blood samples were collected from children recruited into the three major malaria-based studies in KHRC. The recruitment processes were done with the support of the established Health Demographic Surveillance System (KHRC-HDSS). Capillary blood samples by finger-prick were collected from the participants on their study visit days and on any other days participants had fever or an axillary temperature greater or equal to 37.5 °C, for a period stated and approved by the various study protocols, and were referred for further treatment and management. Samples were collected into 0.5 ml microtainers ethylenediaminetetracetic acid (K2EDTA-BD, USA) and were transported to the clinical laboratory in the research centre in Kintampo for analysis.

## Laboratory procedure

### Staining of thick and thin smears

Two blood slides were prepared for each sample that came to the laboratory. Each slide had a measured volume of 6 μl of blood for thick film and 2 μl for the thin film. Ten percent (1:9 ml) for 10 min and 3 % (3:97 ml) for 45–60 min fresh, working Giemsa stains were prepared with already prepared stock of Giemsa-staining solution and working Giemsa buffer prepared from buffer tablets. Thin and thick blood smear were stained with Giemsa after fixing the thin smear with absolute methanol. The 10 % Giemsa stain was used to stain one of the two slides for preliminary slide reading to release results for participant management and treatment. The slide stained with 3 % was given out by the slide coordinator to two competent, independent malaria microscopists. A positive smear was included with each new batch of working Giemsa stain for quality control.

### Examination of thick/thin smears

The entire smear was first screened at a low magnification (10X × 40X objective lens) to detect suitable fields with even distribution of WBC (10–20 WBC/field) [9,12]. Smears were then examined using X100 oil immersion. At least 100 high power fields were examined before a thick smear was declared negative. *Plasmodium falciparum* parasites were counted per 200 or 500 leukocytes, which were used to estimate the parasite density per microlitre of blood. Thin films were examined to confirm the species identification on the thick film. Blood slide was declared positive when a concordant result was produced by two competent microscopists.

### Haematological analysis

The full blood counts (FBC) analysis for each participant’s sample was analysed using the ABX Micros 60 (Horiba ABX, Montpellier, France). Daily internal quality controls and the scheduled external quality assessment programme were adhered to as a quality measure. Two results for all analysed samples were printed. One was filed in the laboratory and the other dispatched into participant’s folder.

### Estimation of parasite density

Parasite densities were recorded as a ratio of parasites to WBC in thick films. *Plasmodium* parasites were counted against 200 WBC on the thick film. Five hundred WBC were counted where less than nine parasites were counted after counting against 200 WBC. Where microscopists did the parasite counts in the thin film (against 2,000 red blood cells) as a result of heavy parasitaemia (greater or equal to 100 parasites per thick smear high power field), parasites counted were recalculated with 200 WBC. Parasite densities (parasite/μl of whole blood) were then calculated as follows:

= (Number of parasites counted/WBC counted) × WBC count/ μL of participant.

Also, parasite densities for all participants were calculated using assumed WBC of 5.0 × 10(9)/L, 6.0 × 10(9)/L, 8.0 × 10(9)/L and 10.0 × 10(9)/L of blood; all set by WHO to be used conveniently in facilities which lack the tools to determine patients’ absolute FBC values.

**Figure 1:**
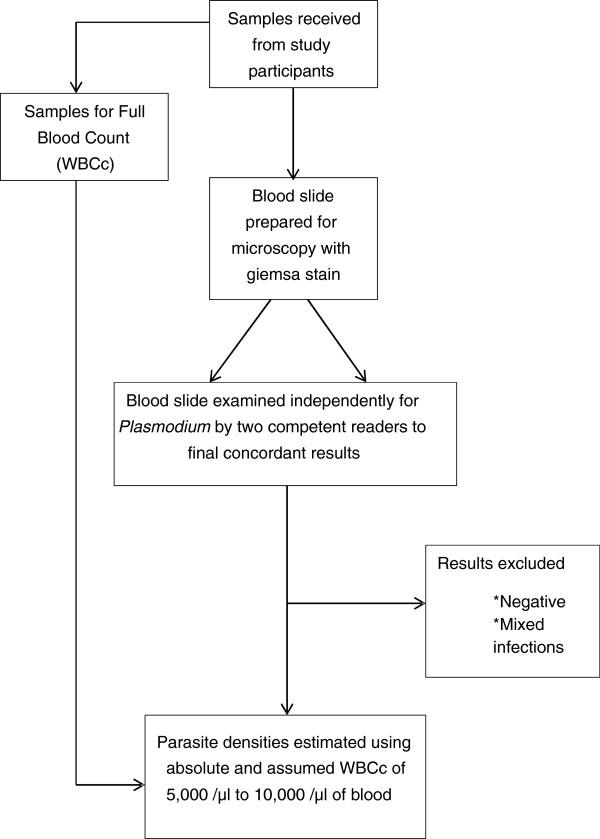
**Flow chart of sample processing and results analysis for*Plasmodium falciparum*density estimation.**

The summary of the methods employed for sample collection and laboratory analyses is illustrated in Figure 1.

### Data entry, cleaning and analyses

Data was checked for completeness and consistency and all queries resolved after double data entry. All negative blood slide results were removed from the data set for analysis. Clean data were analysed using the statistical programs STATA (version 11; Stata Corp., TX USA) and GraphPad PRISM version 5.0 (GraphPad Software, Inc). P-values under 0.05 were considered significant and 95 % CI of geometric means that did not overlap was considered a significant difference.

## Results

Of the 33,540 results received over the study period, 5,902 participant results, that indicated mono-infection with *P. falciparum,* were used for the analysis. All other results that were excluded from analysis were either negative or had mixed infection.

From the 5,902 *P. falciparum* malarial positive samples, using the absolute WBCc of the participants, the mean (SD) WBCc and geometric mean parasite density were 10.4 (4.6) × 10(9)/L and 7557/μL (95 % CI 7144/μL to 7994/μL) of blood respectively. Using an assumed WBCc of 5.0 × 10(9)/L to calculate the parasite density for each participant gave a geometric mean of 3937/μL (95 % CI, 3723/μL to 4164/μL) of blood. For assumed WBCc of 6.0 × 10(9)/L, a geometric mean of parasite density of 4725/μL (95 % CI, 4467/μL to 4997/μL) of blood was obtained. For 8.0 × 10(9)/L, a geometric mean of parasite density of 6300/μL (95 % CI, 5956/μL to 6663/μL) of blood and for assumed WBCc of 10.0 × 10(9)/L; geometric mean parasite density of 7874/μL (95 % CI, 7445/μL to 8328/μL) of blood was estimated (Table 1).

**Table 1:** Comparison of parasite densities using absolute WBCc and assumed WBCc

	**Parasite density with** **Abs. WBCc**	**Parasite density with WBCc of**	**Parasite density with WBCc of**	**Parasite density with WBCc of**	**Parasite density with WBCc of**
		**5.0 × 10(9) /L**	**6.0 × 10(9) /L**	**8.0 × 10(9) /L**	**10.0 × 10(9) /L**
**Number of observations,*N***	5,902	5,902	5,902	5,902	5,902
**Minimum**	15	10	12	16	20
**25 % Percentile**	1,479	750	900	1,200	1,500
**Median**	7,815	4,150	4,980	6,640	8,300
**75 % Percentile**	40,420	21,275	25,530	34,040	42,550
**Maximum**	4,036,350	1,940,553	2,328,663	3,104,885	3,881,106
**Mean**	49,474	26,327	31,592	42,123	52,654
**Std. Deviation**	122,009	72,417	86,900	115,867	144,834
**Std. Error**	1,588	943	1,131	1,508	1,885
**Lower 95 % CI of mean**	46,360	24,479	29,375	39,167	48,958
**Upper 95 % CI of mean**	52,587	28,175	33,810	45,080	56,350
**Geometric mean**	7,557	3,937	4,725	6,300	7,874
**Lower 95 % CI of geo. Mean**	7,144	3,723	4,467	5,956	7,445
**Upper 95 % CI of geo. Mean**	7,994	4,164	4,997	6,663	8,328

The difference in the geometric mean parasite densities calculated using absolute WBCc compared to densities estimated with assumed WBCc were significantly lower for 5.0 × 10(9)/L, 6.0 × 10(9)/L and 8.0 × 10(9)/L of blood. However, geometric mean parasite density for assumed WBCc of 10.0 × 10(9)/L estimated a geometric mean parasite density of 7874/μL of blood that at 95 % CI, 7445/μL to 8328/μL of blood was not significantly different from using the absolute WBCc of the participants.

## Discussion

Estimating malaria parasite density is necessary for patient management especially when parasite resistance to available therapy is on the increase and particularly in clinical trials and drug efficacy studies. This has become dominated by the convenient but inaccurate assumption of a constant WBCc of 8.0 × 10(9)/L of peripheral blood [14] set by WHO to be used for parasite density estimation.

It is agreed that the best solution in estimating parasite densities would be to use the corresponding standard WBCc for each age group [15] but since most laboratories in malaria-endemic countries in sub-Saharan Africa may not have the capacity to measure the WBCc [16] of patients for parasite density estimation, using a standardized WBCc would be acceptable. Estimating the parasite density of *P. falciparum* with the standardized WBCc of 8000/ul of blood [9] set by WHO, would mean underestimating significantly the parasite density of *P. falciparum* infections for patients in communities such as Kintampo North, South and Wenchi Municipality. Therefore, establishing regional-based, standard WBCc as suggested by Trape (1985) to estimate parasite densities in malarial infections will be appropriate. The geometric mean parasite density, 7,874/μL of blood, estimated by using assumed WBCc of 10,000/ul of blood at 95 % CI (7,445/μL to 8,328/μL of blood) overlaps with the geometric mean parasite density obtained by using the absolute WBCc; 7,557/μL (95 % CI 7,144/μL to 7,994/μL) of blood, of the participants, while the parasitaemia obtained by the use of the other assumed WBCc were significantly lower.

However, clinical trials of uncomplicated falciparum malaria and drug efficacy studies should express parasitaemia calculated on the basis of the patient’s own WBCc since using actual WBCc will increase precision in estimating parasitaemia and improve our understanding of malaria and response to treatment [2] and changes that might need peculiar attention for management.

### Limitations

The ages of all participants were not stratified into groups. This did not make it possible to compare differences at the possible age groups less than five years. Information on sexes was not included for analysis.

## Conclusions

In this study in Central Ghana involving sick and healthy children less than five years, the mean WBCc was 10.4 × 10(9)/L, which is within the range of 4.0–11 × 10(9)/L accepted worldwide [17]. The study proposes the use of standardized WBCc in malaria-endemic regions for determining *P. falciparum* parasitaemia or when such standard values are not available, assumed WBCc of 10.0 × 10(9)/L, rather than WBCc of 8.0 × 10(9)/L of blood set by WHO, should be used to obtain precise density estimation. The study suggests that the WBCc of individuals should be measured to estimate parasite density, especially in drug efficacy and clinical trials.

## Competing interests

The authors declare that they have no competing interests.

## Authors' contributions

DA-G designed the study, performed most of the experiments and wrote the manuscript. MA and SA contributed to the study design, performance of experiments and wrote the manuscript. EM assisted with study design and carried out the statistical analysis. MN and SA-E contributed to the study design, performance of experiments and data analysis. KPA, SN, FB and SO-A designed the experiments, supervised the study and wrote the manuscript. All authors read and approved the final version of the manuscript.

## References

[B1] Sachs J Malaney P The economic and social burden of malaria Nature 2002 415 680 685 10.1038/415680a 11832956

[B2] Jeremiah ZA Uko EK Comparative analysis of malaria parasite density using actual and assumed white blood cell counts Ann Trop Paediatr 2007 27 75 79 10.1179/146532807X170547 17469736

[B3] Karyana M Burdarm L Yeung S Kenangalem E Wariker N Maristela R Umana KG Vemuri R Okoseray MJ Penttinen PM Malaria morbidity in Papua Indonesia, an area with multidrug resistant Plasmodium vivax and Plasmodium falciparum Malar J 2008 7 148 10.1186/1475-2875-7-148 18673572 PMC2518158

[B4] McKenzie FE Prudhomme WA Magill AJ Forney JR Permpanich B Lucas C Gasser RA Wongsrichanalai C White blood cell counts and malaria J Infect Dis 2005 192 323 10.1086/431152 15962228 PMC2481386

[B5] O'Meara WP Barcus M Wongsrichanalai C Muth S Maguire JD Jordan RG Prescott WR McKenzie FE Reader technique as a source of variability in determining malaria parasite density by microscopy Malar J 2006 5 118 10.1186/1475-2875-5-118 17164007 PMC1712346

[B6] Van den Bossche J Devreese K Malfait R Van de Vyvere M Wauters A Neels H De Schouwer P Reference intervals for a complete blood count determined on different automated haematology analysers: Abx Pentra 120 Retic, Coulter Gen-S, Sysmex SE 9500, Abbott Cell Dyn 4000 and Bayer Advia 120 Clin Chem Lab Med 2002 40 69 73 11916274 10.1515/CCLM.2002.014

[B7] Briggs C Harrison P Machin SJ Continuing developments with the automated platelet count Int J Lab Hematol 2007 29 77 91 10.1111/j.1751-553X.2007.00909.x 17474881

[B8] Petersen E Marbiah NT New L Gottschau A Comparison of two methods for enumerating malaria parasites in thick blood films AmJTrop Med Hyg 1996 55 485 489 10.4269/ajtmh.1996.55.485 8940978

[B9] WHO W.H.O., Basic Malaria Microscopy 2010 http://www.searo.who.int/LinkFiles/Malaria_malaria_microscopy_Learners_guide2010.pdf

[B10] Planche T Krishna S Kombila M Engel K Faucher JF Ngou-Milama E Kremsner PG Comparison of methods for the rapid laboratory assessment of children with malaria AmJTrop Med Hyg 2001 65 599 602 10.4269/ajtmh.2001.65.599 11716121

[B11] O'Meara WP Hall BF McKenzie FE Malaria vaccine efficacy: the difficulty of detecting and diagnosing malaria Malar J 2007 6 36 10.1186/1475-2875-6-36 17386083 PMC1852320

[B12] Owusu-Agyei S Asante KP Adjuik M Adjei G Awini E Adams M Newton S Dosoo D Dery D Agyeman-Budu A Gyapong J Greenwood B Chandramohan D Epidemiology of malaria in the forest-savanna transitional zone of Ghana Malar J 2009 8 10.1186/1475-2875-8-220 PMC2765449 19785766

[B13] Quashie NB Duah NO Abuaku B Koram KA The in-vitro susceptibilities of Ghanaian Plasmodium falciparum to antimalarial drugs Ann Trop Med Parasito 2007 101 391 398 10.1179/136485907X176553 17550644

[B14] Greenwood BM Armstrong JP Comparison of two simple methods for determining malaria parasite density Trans R Soc Trop Med Hyg 1991 85 186 188 10.1016/0035-9203(91)90015-Q 1887466

[B15] Trape JF Rapid evaluation of malaria parasite density and standardization of thick smear examination for epidemiological investigations Trans R Soc Trop Med Hyg 1985 79 181 184 10.1016/0035-9203(85)90329-3 3890280

[B16] Piero O Abdoulaye D Corine K Andreas M Jean-Louis N Sodiomon BS Grant D Julien Z Standardised versus actual white cell counts in estimating thick film parasitaemia in African children under five Trop Med Int Health 2011 16 551 554 10.1111/j.1365-3156.2011.02738.x 21395926

[B17] Brown BA Hematology: Principles and Procedures 1993 6 Lea & Febiger, Philadelphia, PA

